# New opportunities for time-resolved imaging using diffraction-limited storage rings

**DOI:** 10.1107/S1600577524005290

**Published:** 2024-07-30

**Authors:** Zisheng Yao, Julia Rogalinski, Eleni Myrto Asimakopoulou, Yuhe Zhang, Korneliya Gordeyeva, Zhaleh Atoufi, Hanna Dierks, Samuel McDonald, Stephen Hall, Jesper Wallentin, Daniel Söderberg, Kim Nygård, Pablo Villanueva-Perez

**Affiliations:** a Synchrotron Radiation Research and NanoLund, Department of Physics Lund University Lund Sweden; b Department of Fibre and Polymer Technology Royal Institute of Technology Stockholm Sweden; c MAX IV Laboratory Lund University Lund Sweden; d Lund Institute of Advanced Neutron and X-Ray Science (LINXS), Lund, Sweden; University of Malaga, Spain

**Keywords:** time-resolved microscopy, MAX IV, ForMAX beamline, megahertz imaging, diffraction-limited storage rings

## Abstract

The advent of diffraction-limited storage rings (DLSRs) has boosted the brilliance or coherent flux by one to two orders of magnitude with respect to the previous generation. One consequence of this brilliance enhancement is an increase in the flux density or number of photons per unit of area and time, which opens new possibilities for the spatiotemporal resolution of X-ray imaging techniques. This paper studies the time-resolved microscopy capabilities of such facilities by benchmarking the ForMAX beamline at the MAX IV storage ring. It is demonstrated that this enhanced flux density using a single harmonic of the source allows micrometre-resolution time-resolved imaging at 2000 tomograms per second and 1.1 MHz 2D acquisition rates using the full dynamic range of the detector system.

## Abstract 2

The opportunities for time-resolved X-ray imaging at diffraction-limited storage rings are demonstrated by benchmarking time-resolved 2D and 3D imaging at ForMAX, MAX IV. The enhanced flux density provided by the MAX IV storage ring allows micrometer-resolution time-resolved imaging at 2 kHz and 1.1 MHz acquisition rates in 3D and 2D, respectively.

## 1. Introduction

Over the past few decades, the availability of and advances in synchrotron radiation X-ray sources have boosted the field of X-ray imaging. Specifically, the advent of diffraction-limited storage rings (DLSRs), the latest generation of storage rings, has opened new possibilities for X-ray imaging. DLSRs utilize multi-bend achromats (MBAs), resulting in one to two orders of magnitude increase in the brilliance or coherent flux (Eriksson, 2016 ▸; Raimondi & Liuzzo, 2023 ▸). Such a brilliance increase leads to a proportional increase in flux density, *i.e.* the number of photons per unit of area and time. Currently, three DLSRs (MAX IV in Sweden, SIRIUS in Brazil and ESRF-EBS in France) are in operation (Eriksson *et al.*, 2014 ▸; Liu *et al.*, 2014 ▸; Raimondi *et al.*, 2023 ▸) and several more are under construction (Yabashi & Tanaka, 2017 ▸; Streun *et al.*, 2018 ▸; Samuel Reich, 2013 ▸; He *et al.*, 2023 ▸).

**Figure 1:**
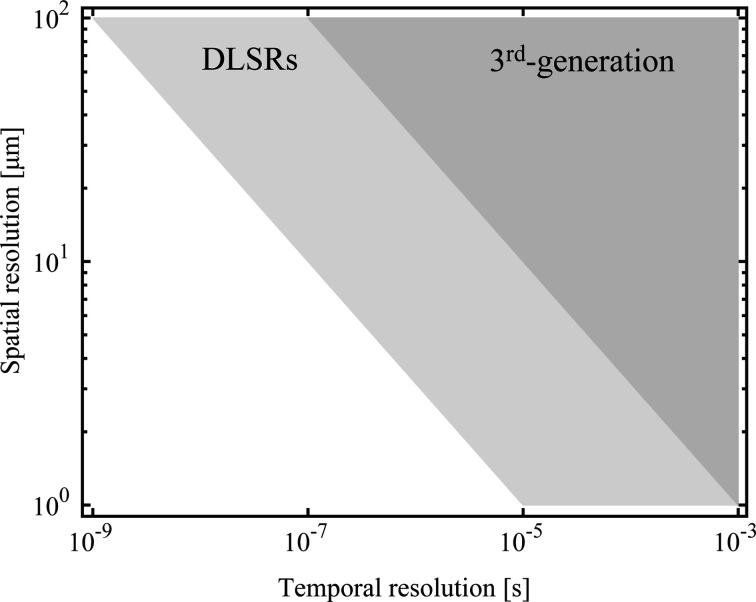
A comparison of spatiotemporal resolution between DLSR-based synchrotron light sources and third-generation synchrotron light sources. It is based on the relationship ϕ = *N*/(τΔ*x*^2^), where ϕ denotes the flux density, *N* denotes the number of photons required to achieve a certain image quality, τ denotes the temporal resolution and Δ*x* denotes the spatial resolution. The typical values we use for the flux density at DLSRs and third-generation synchrotron light sources are 10^15^ photons s^−1^ mm^−2^ and 10^13^ photons s^−1^ mm^−2^ (Nygård *et al.*, 2024 ▸; Willmott, 2019 ▸), respectively. We further assume that *N* = 10^4^, as it leads to a signal-to-noise ratio of *N*/(*N*^1/2^) = 100 (Poisson statistics), which can provide high-quality images for most imaging experiments.

With the increased flux density of modern DLSR-based light sources, X-ray beams can now provide improved spatiotemporal resolution for imaging techniques, allowing us to push the limits of time-resolved imaging. Fig. 1 ▸ illustrates a comparison of typical spatiotemporal resolution between DLSRs and third-generation synchrotron light sources. It shows that we can enhance the temporal resolution up to one hundred times for a given sample while retaining the same spatial resolution compared to the previous generation of storage rings (Eriksson *et al.*, 2014 ▸; Eberhardt, 2015 ▸). This means that, although the trade off among spatial resolution, temporal resolution and sample contrast still exists (Russo, 2017 ▸; Bhandari *et al.*, 2022 ▸), we can study faster phenomena without compromising on the other factors for a given sample studied at a fixed energy. When it comes to X-ray imaging, there is another trade off to consider between DLSRs and the previous generation of storage rings. Although DLSRs do not typically produce more X-ray photons than their predecessors, they effectively reduce the phase space and hence increase the flux density. This trade off results in compromising the sample volume that can be studied with enhanced spatiotemporal resolution as the beam becomes smaller. In the context of this work, we focus on studying the time-resolved imaging capabilities of DLSRs at the micrometre scale and the new opportunities that arise from this enhanced flux density.

Time-resolved X-ray imaging is a tool that enables non-destructive studies under *in situ* and *operando* conditions of phenomena of relevance for a plethora of disciplines, such as fluid dynamics (Fezzaa & Wang, 2008 ▸; Tekawade *et al.*, 2020 ▸), additive manufacturing (Zhao *et al.*, 2022 ▸; Makowska *et al.*, 2023 ▸) and fast biological processes (Levantino *et al.*, 2015 ▸; Truong *et al.*, 2020 ▸). Such scientific cases may require either time-resolved 2D or 3D imaging approaches. State-of-the-art time-resolved 2D imaging can achieve megahertz frame rates with micrometre resolution in synchrotron light sources (Escauriza *et al.*, 2018 ▸; Wang *et al.*, 2018 ▸; Xie *et al.*, 2019 ▸; Rack, 2020 ▸). Various scientific cases have been investigated. For example, pulsed water plasma events were recorded with a frame rate of 0.3 MHz and a spatial resolution of 2 µm (Campbell *et al.*, 2021 ▸), and crack propagation in glass was recorded with a frame rate of 1.4 MHz and a spatial resolution of 8 µm (Olbinado *et al.*, 2017 ▸). Such time-resolved 2D imaging can be operated in either single-pulse mode (Luo *et al.*, 2012 ▸), using the exposure from a single synchrotron X-ray pulse, or multi-pulse mode (Gao *et al.*, 2022 ▸), *i.e.* integrating over several X-ray pulses. The single-pulse mode allows for observation of transient processes, while the multi-pulse mode allows access to a higher average flux density (Olbinado & Rack, 2019 ▸). In this work, we focus on the multi-pulse mode, as MAX IV is a small storage ring with a high pulse frequency of 100 MHz.

Time-resolved 3D X-ray imaging based on tomography provides one of the highest temporal resolutions together with micrometre resolution. It relies on a continuous and fast rotation stage that acquires projections at different angles between the X-ray beam and the sample. Assuming that we have sufficient flux to retrieve a given spatial resolution for a specific sample contrast (Villanova *et al.*, 2017 ▸; Garcia-Moreno *et al.*, 2023 ▸), faster rotations can lead to faster 3D movies. Advanced rotational stages together with high-flux sources have demonstrated time-resolved 3D imaging with a frame rate of up to 1000 tomograms per second (tps) (Garcia-Moreno *et al.*, 2021 ▸) coupled with a spatial resolution of 8.2 µm. This breakthrough was achieved at TOMCAT (SLS, Switzerland), using the broadband spectrum provided by a super-bending magnet. Such broadband provides a high integrated flux over a broad spectrum, but it hinders quantitativeness as it may introduce artifacts such as beam hardening (Brooks & Di Chiro, 1976 ▸). An alternative to this is to use an insertion device with a narrow single harmonic as provided by undulators (Δλ/λ ≈ 10^−2^), but at the expense of temporal resolution due to a smaller flux than with a white beam. In this work, we exploit a single harmonic (third order at 9.1 keV, fifth order at 12.7 keV or seventh order at 16.3 keV) filtered by a multilayer monochromator to evaluate the time-resolved imaging opportunities at MAX IV.

In this study, we evaluate the time-resolved imaging capabilities of ForMAX (Nygård *et al.*, 2024 ▸), a newly established imaging beamline at MAX IV. We present the potential to acquire images with micrometre resolution at 1.1 MHz and 2000 tps acquisition rates with time-resolved 2D and 3D imaging, respectively. As a result of the high flux density, we demonstrate that such high acquisition rates can be obtained using the full dynamic range (12 bits) of our detector system. With this bit depth, the sample contrast sensitivity increases compared to optical systems with smaller bit depths. Such results illustrate the capabilities of time-resolved imaging at DLSRs as a result of the unprecedented flux density provided by such facilities.

The paper is structured as follows. First, we introduce the instrumental setup on the ForMAX beamline. Second, we describe the sample and sample environment used in the presented experiments. Third, we present the 2D and 3D results and evaluate the resolution of the recorded images. Finally, we discuss the obtained results and give an outlook for possible applications and future experiments.

## 2. Instrumentation

MAX IV, located in Lund, Sweden, is the first operational DLSR-based facility. It is based on a 3 GeV storage ring with a circumference of 528 m, supplied with a ring current of 400 mA. Operating at a maximum injection repetition rate of 10 Hz and an RF frequency of 100 MHz, this storage ring delivers an electron beam with remarkably low emittance ε of 326 pm rad × 8 pm rad (H × V) (Tavares *et al.*, 2014 ▸), setting the diffraction limit in the horizontal direction to 4 nm (Willmott, 2019 ▸).

### 2.1. Beamline configuration

The ForMAX beamline at MAX IV provides multiscale structural characterization, with a special focus on research on forest materials. Therefore, it is dedicated to studying samples such as wood and fibers in the nanometre to millimetre range. This beamline is optimized for X-ray scattering and imaging experiments, and employs techniques such as small- and wide-angle X-ray scattering (SWAXS) and tomography (Nygård *et al.*, 2024 ▸).

The synchrotron radiation on ForMAX is generated by a 3 m long in-vacuum undulator with a period length of 17 mm (IVU-17) and a *K* value (maximum effective deflection parameter) of 1.89 (at the minimum gap). The produced X-ray energies range from 8 keV to 25 keV, and the desired energy can be selected using either a double-crystal monochromator (DCM) or a double-multilayer monochromator (MLM). In our experiment, we chose the MLM [beam size ∼1.3 mm (H) × 1.5 mm (V)] to achieve a higher photon flux compared with the DCM. The MLM accepts an entire single harmonic provided by the undulator (Nygård *et al.*, 2024 ▸).

**Figure 2:**
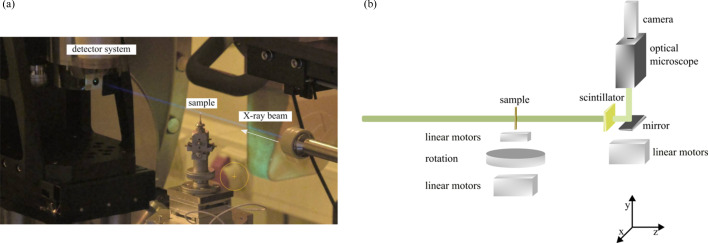
The experimental setup of ForMAX. (*a*) X-rays ionize the air, leading to fluorescence in the NUV wavelength spectrum as visualized by an Axis camera surveying the beamline. (*b*) Schematics of the setup. The X-rays pass through the sample and are converted into visible light. The visible light is deflected upwards and passes through an optical microscope for magnification before it is detected by a high-speed camera.

We operated the undulator with the smallest gap to achieve the highest flux density that ForMAX can provide. This fixed the studied energies to 9.1 keV (third harmonic), 12.7 keV (fifth harmonic) and 16.3 keV (seventh harmonic). The high photon flux of the order of 10^15^ photons s^−1^ at 9.1 keV can be seen vividly as the violet glow of the beam in Fig. 2 ▸(*a*): given the large number of photons per second, the nitrogen in the air is ionized, resulting in fluorescence upon deexcitation with dominant emission occurring in the near-ultraviolet (NUV) wavelength spectrum (300–400 nm) (Chichester & Watson, 2011 ▸). Having a high flux density is advantageous for performing time-resolved imaging as it scales linearly with temporal resolution.

### 2.2. Experimental setup

We performed our experiments using a standard tomography setup with a rotation stage from LAB Motion Systems (RT075s) (Dierks *et al.*, 2023 ▸), as depicted in Fig. 2 ▸(*b*). To acquire the images, we employed an indirect detector system, comprising a scintillator, an optical microscope and a high-speed camera. We used a 250 µm-thick scintillator (GAGG+), matching the depth of focus for our desired resolution (∼8 µm), to convert the X-rays into visible light, followed by an optical microscope equipped with a 5× infinity-corrected objective and a 1× tube lens from Mitutoyo. Signal detection was accomplished using a Photron Nova S16 high-speed camera with a physical pixel size of 20 µm (corresponding to an effective pixel size of 4 µm). The number of pixels varied with the acquisition rate. One can use up to 1024 × 1024 pixels and an acquisition rate of up to 16 kHz. However, faster acquisition rates than 16 kHz can decrease the maximum number of pixels available with a continuous-readout image sensor (Mittone *et al.*, 2017 ▸) like the detector used here. Specifically, a Photron Nova S16 provides 128 × 64 and 128 × 16 pixels at frame rates of 550 kHz and 1.1 MHz, respectively. This is shown in Figs. 4(*a*) and 4(*c*). To avoid reducing the number of pixels as a function of acquisition rate, one can alternatively use fast cameras based on a burst image sensor with pixel-based storage (Olbinado *et al.*, 2017 ▸), *e.g.* a Shimadzu HPV-X2.

## 3. Sample and sample environment

The samples we investigated were cellulose filaments, cellulose foams and wooden rods. An overview of the studied samples and dynamic processes with the corresponding experimental parameters is given in Table 1 ▸. We performed all measurements at room temperature and under ambient pressure. For our 2D experiments, we used a loading stage (maximum force 9.8 N) (Pettersson *et al.*, 2019 ▸) on top of the stack of motors to expose the samples to external forces (contraction or extension), allowing us to capture their dynamics.

Cellulose nanofibrils (CNFs) are nanoparticles extracted from wood. There is extensive research ongoing to establish CNFs as a renewable and biodegradable natural matrix for materials such as foams and filaments. We have previously developed a technique for the production of the strongest bio-based filaments using microfluidic devices with focusing flows (Mittal *et al.*, 2017 ▸). Outstanding mechanical properties are achieved by efficient alignment of CNFs by extensional forces and rapid locking of the aligned system by charge suppression of negative groups on the CNF surface. Such filaments can be a perfect matrix for forming advanced functional materials for such applications as wearable devices which are playing a crucial role in healthcare, especially for patients in a critical condition. Therefore, it is of high relevance to understand their behavior under mechanical stress. The filaments examined in this work had a diameter of 3–12 µm, making up multi-filaments of cylindrical shape with an oblate cross section (width of overall cross section 250–300 µm). The vertical dimension of the samples was roughly 10 mm.

One of the key needs for developing cellulose-based foams lies in the rising demand for replacing fuel-based filters for the purification of water from oils and heavy metals with natural alternatives. In this work, heat-treated beta-lactoglobulin was used as a green surfactant and was prepared from whey protein (Adamcik & Mezzenga, 2012 ▸), a by-product of cheese production. CNFs played the role of a wet foam visco-elastic stabilizer (Gordeyeva *et al.*, 2016 ▸). By manipulating electrostatic interactions and/or inducing covalent cross-linking between heat-treated beta-lactoglobulin and CNFs a re­inforced interconnected matrix could be formed. Each manipulation will have a different impact on the pore morphology and structure (size, shape, wall thickness) and can therefore heavily affect the foam’s mechanical properties. Here, we studied foams with a cylindrical shape and a diameter of approximately 1 mm. The length of the samples was about 10 mm, matching our tensile setup gap.

One of the caveats of imaging wood, and biological samples in general, with X-rays is radiation damage. When imaging a sample with a high flux density over extended periods of time, we may introduce structural changes that can hinder the retrieved resolution (Howells *et al.*, 2009 ▸). Thus, mitigation strategies such as reducing the exposure time or using cryo-options must be contemplated. To illustrate such an effect as a function of exposure time under a high flux density, we studied a wooden rod [2 mm × 2 mm × 50 mm (l × w × h)].

## 4. Results

### 4.1. Evaluation of the spatial resolution (two dimensions)

**Figure 3:**
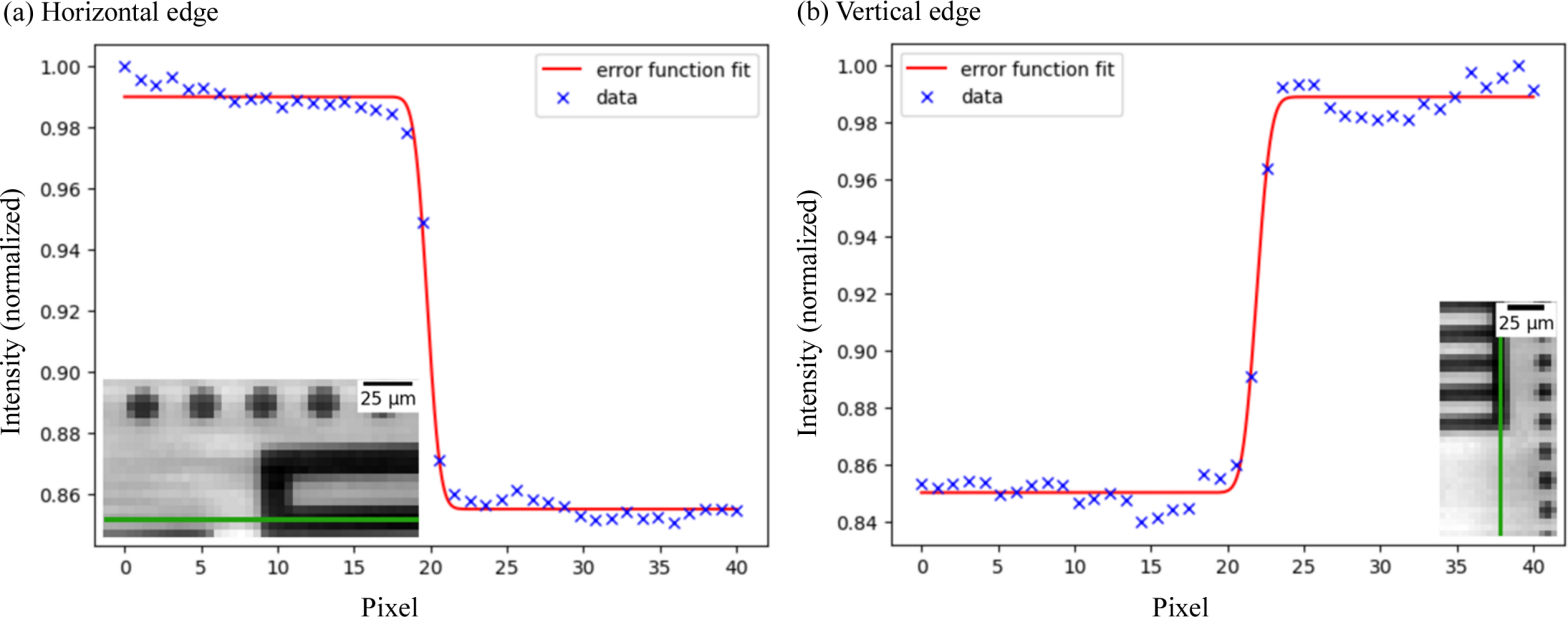
Recorded images of a JIMA RT RC-02B resolution chart to assess the spatial resolution of the detector system in two dimensions. The analyzed edges are marked in green, and the extracted intensities with their corresponding error function fits are shown for (*a*) a horizontal edge and (*b*) a vertical edge.

To assess the spatial resolution we can achieve with our overall detector system in two dimensions, we recorded images of the JIMA RT RC-02B resolution chart (energy 16.3 keV, Si filters with a thickness of 1.5 mm, acquisition rate 40 kHz), as depicted in Fig. 3 ▸. The optical elements influence the achievable spatial resolution by introducing a point spread function (PSF). The measured image is then the convolution between the actual object and the PSF (Ohkubo *et al.*, 2009 ▸). This convolution results in a loss of sharpness to a certain extent in the final image, motivating an analysis of the overall system resolution.

To evaluate the horizontal and vertical resolution, the analysis involves examining the intensity transition along both a horizontal and a vertical edge. The selected edges, depicted as green bars in Fig. 3 ▸, are each 40 pixels long and 1 pixel wide. By extracting the intensity values along these edges, we obtained the intensity profile along the edge. A rapid transition indicates a sharp well resolved edge. To assess the transition, the intensity profile was fitted with an error function, from which the corresponding standard deviation σ was derived. This value was used to compute the FWHM (= 2.35σ), providing insights into the intensity variation along the edge (Shinohara & Hashimoto, 2022 ▸; Amorese *et al.*, 2019 ▸). The horizontal (vertical) intensity profile and corresponding fit are illustrated in Fig. 3 ▸(*a*) [Fig. 3 ▸(*b*)].

We determined the standard deviations of the horizontal and vertical edges to be 0.67 ± 0.06 pixels and 0.73 ± 0.09 pixels, respectively. Considering an effective pixel size of 4 µm, this translates to approximately 2.68 µm (horizontal) and 2.92 µm (vertical). The corresponding FWHMs were then calculated to be 6.30 µm and 6.86 µm. However, according to the Nyquist–Shannon theorem a signal, *e.g.* the spatial frequency in an image, can only be restored if the sampling rate is at least twice the highest spatial frequency in the image (Thapa *et al.*, 2015 ▸). Therefore, it is essential to note that in practical terms, the spatial resolution of our system is constrained to 2 × 4 µm = 8 µm.

### 4.2. Time-resolved 2D imaging

#### 4.2.1. Evaluation of dynamic range

To evaluate the speed and the corresponding dynamic range at which we can record images with our detector system, we set the X-ray energy to 12.7 keV and 16.3 keV. We estimated that the photon flux was 1 × 10^15^ photons s^−1^ at 12.7 keV and 5 × 10^14^ photons ^−1^ at 16.3 keV (Nygård *et al.*, 2024 ▸). Thus, the corresponding beam powers were 1.04 W mm^−2^ at 12.7 keV and 0.67 W mm^−2^ at 16.3 keV. We further estimated that the surface dose rates (Howells *et al.*, 2009 ▸) on our scintillator were 1.15 × 10^7^ Gy s^−1^ and 3.77 × 10^6^ Gy s^−1^ at 12.7 keV and 16.3 keV, respectively.

**Figure 4:**
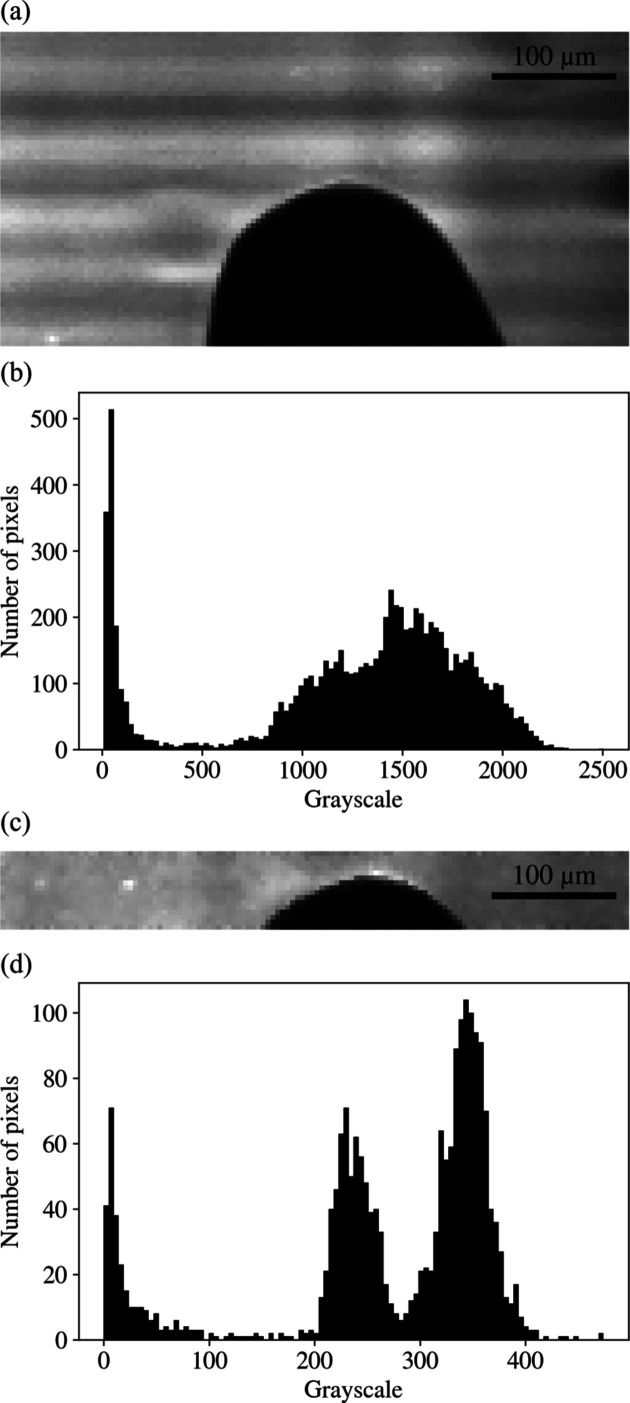
Dynamic range evaluation. (*a*) An image of a static metal pin recorded at 550 kHz at 16.3 keV and (*b*) the corresponding histogram. (*c*) An image of a static metal pin recorded at 1100 kHz at 12.7 keV and (*d*) the corresponding histogram.

The sample we used was a static metal pin. The results at 16.3 keV are shown in Figs. 4 ▸(*a*) and 4 ▸(*b*). We used the full X-ray beam intensity. The maximum and minimum grayscales of the image shown in Fig. 4 ▸(*b*) were 2504 and 8, respectively. Since 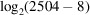 ≈ 11.3, the dynamic range was 12 bits at a frame rate of 550 kHz.

Another evaluation was made at an energy of 12.7 keV, which is shown in Figs. 4 ▸(*c*) and 4 ▸(*d*). To prevent possible damage to the scintillator because of the larger photon flux at this energy, we used a total of 600 µm Si filters, corresponding to a transmission rate of 0.109. Under these settings, the maximum and minimum grayscales of the image shown in Fig. 4 ▸(*c*) were 474 and 0, respectively. Since 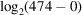 ≈ 8.9, the dynamic range of the image shown in Fig. 4 ▸(*c*) was 9 bits at a frame rate of 1.1 MHz. If we remove all the Si filters and utilize the full beam intensity, we have the potential to use the full dynamic range of our detector (12 bits) at a frame rate of 1.1 MHz.

#### 4.2.2. Sample measurements

Here, we demonstrate two examples of time-resolved 2D imaging, where rapid dynamics can be observed. For these measurements, we set the energy to 16.3 keV and the acquisition rate to 36 kHz (Table 1 ▸). The energy and the frame rate were selected to provide a good contrast for the studied samples and to capture the desired dynamics, respectively. We used a total of 1.5 mm Si filters, corresponding to a transmission rate of 0.071, to limit the flux while using the full dynamic range of the detector at 36 kHz.

**Figure 5:**
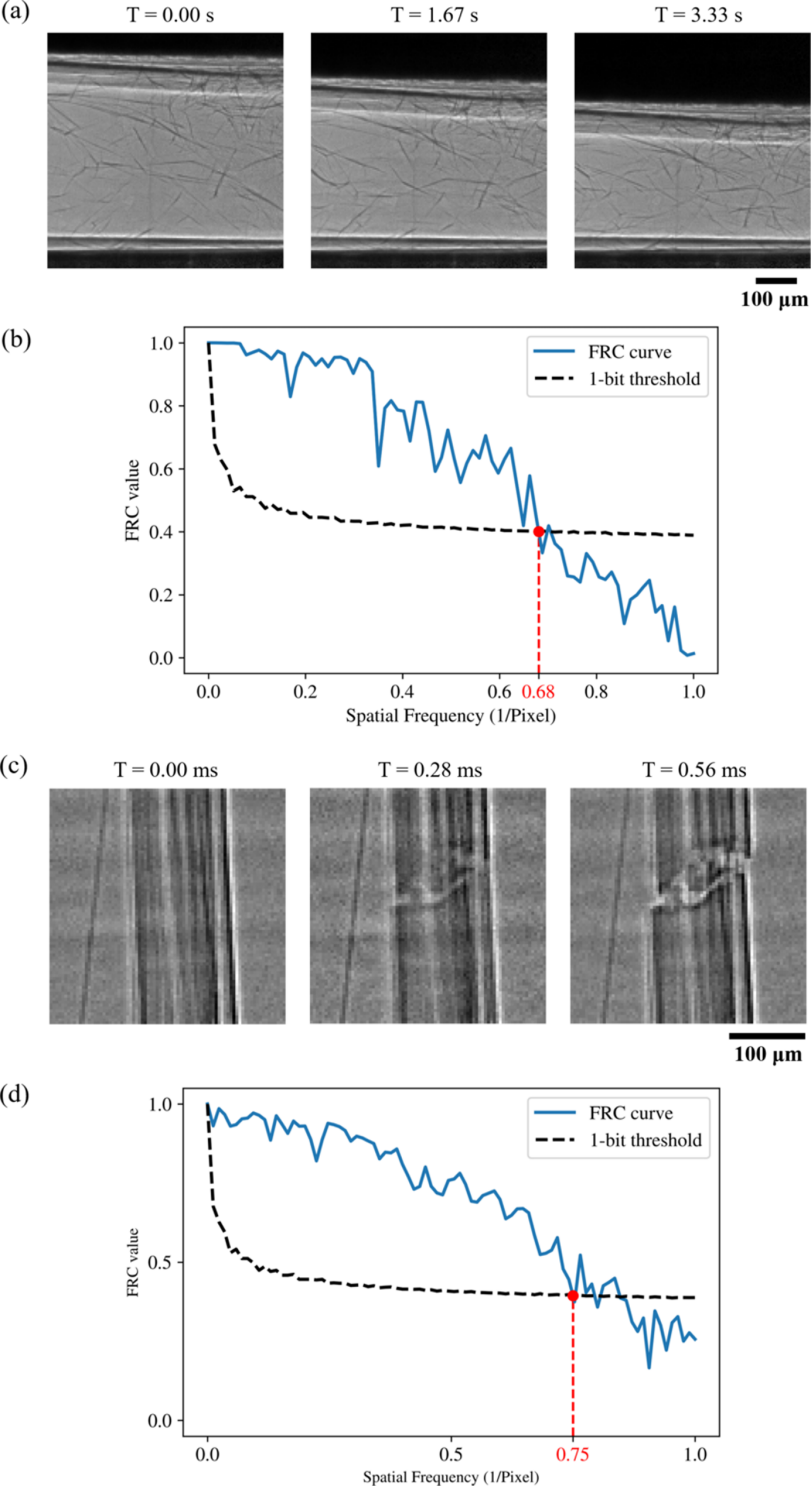
Time-resolved 2D imaging studies. (*a*) Three flat-field corrected frames of the foam compression process and (*b*) FRC analysis of two independent measurements. (*c*) Three flat-field corrected frames of the tensile failure of a multi-filament fiber and (*d*) FRC analysis of two independent measurements. In panels (*b*) and (*d*) the red dots denote the intersection of the FRC curve and the 1 bit threshold curve.

The first example of time-resolved 2D imaging is the foam compression process, which is shown in Figs. 5 ▸(*a*) and 5 ▸(*b*). We exposed the foams to external stress by compressing them at a speed of 2.0 mm min^−1^. Fig. 5 ▸(*a*) shows three flat-field corrected frames during the foam compression process. To analyze the resolution of the 2D sequence, we used Fourier ring correlation (FRC) (Van Heel & Schatz, 2005 ▸), a practical way of estimating the resolution from two independent measurements. According to Fig. 5 ▸(*b*), the FRC curve and the 1 bit threshold curve intersect at *x* = 0.68. Given that the effective pixel size of the images is 4 µm, the resolution is estimated to be (2 × 4 µm)/0.68 = 11.8 µm.

The second example of time-resolved 2D imaging is fracture of a multi-filament fiber, which is shown in Figs. 5 ▸(*c*) and 5 ▸(*d*). We exposed the sample to external tensile stress by loading it at a speed of 0.5 mm min^−1^. Fig. 5 ▸(*c*) shows three consecutive flat-field corrected frames during the fracture process. For this image sequence, we implemented a dynamic flat-field correction based on principal component analysis of the flat-field images (Van Nieuwenhove *et al.*, 2015 ▸; Buakor *et al.*, 2022 ▸; Birnsteinova *et al.*, 2023 ▸). Again, we used FRC to evaluate the resolution of the 2D images. As shown in Fig. 5 ▸(*d*), the FRC curve and the 1 bit threshold curve intersect at *x* = 0.75. Given that the effective pixel size of the images is 4 µm, the resolution is estimated to be (2 × 4 µm)/0.75 = 10.7 µm.

### 4.3. Time-resolved 3D imaging

For the 3D experiments, we set the frame rate at 750 Hz and the X-ray energy at 9.1 keV (Table 1 ▸). We selected this energy to probe the dynamics induced by the radiation damage on the sample due to the high dose and absorption at this energy, as shown in Fig. 2 ▸(*a*). We used a total of 600 µm Si filters with a transmission of 0.0027. The Si filters were placed behind the sample, *i.e.* in front of the scintillator, to be able to induce radiation damage on the sample while ensuring a low flux on the detector. The maximum operational speed of the rotation stage was 180 revolutions per minute, meaning that for every 360° we collected 250 projections of the sample. Under these settings, 6 tomograms per second were acquired, which corresponded to the maximum rotation speed of the stage. This provided us with the full dynamic range (12 bits) of our detector at a frame rate of 750 Hz.

**Figure 6:**
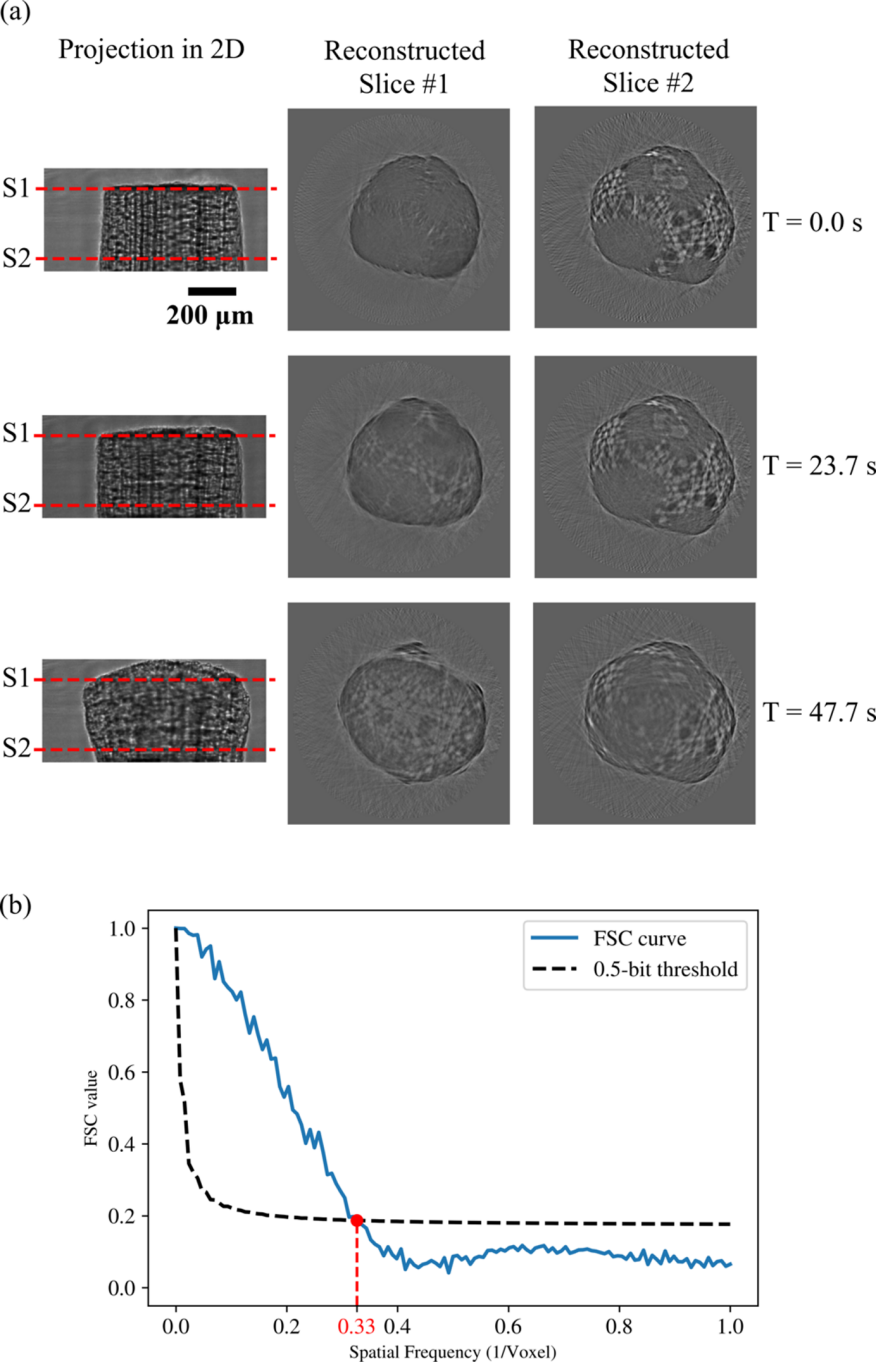
The time-resolved 3D imaging test. (*a*) Flat-field corrected projections and two corresponding reconstructed slices at three different time points during the radiation damage process of a wooden rod. (*b*) FSC analysis of two independent 3D reconstructions of the sample, where the red dot denotes the intersection of the FRC curve and the half-bit threshold curve.

Here, we demonstrate the time-resolved 3D imaging results, showing radiation damage to a wooden rod. For every recorded 2D projection, we implemented flat-field correction by simply dividing the projection image by the empty-beam images, *i.e.* images without the sample in the beam. We then divided the projection dataset into two independent ones and performed tomographic reconstruction with the *Gridrec* algorithm (Marone & Stampanoni, 2012 ▸) in *Tomopy* (Gürsoy *et al.*, 2014 ▸), a Python-based open-source framework. Fig. 6 ▸(*a*) shows the 2D projections and two corresponding reconstructed slices at three different time stamps.

We estimated the resolution of the 3D reconstructions using the Fourier shell correlation (FSC). According to Fig. 6 ▸(*b*), the FSC curve and the 0.5 bit threshold curve intersect at *x* = 0.33. Given that the effective voxel size of the reconstructed object is 4 µm, the resolution is estimated to be (2 × 4 µm)/0.33 = 24.2 µm.

Hence, we have retrieved micrometre resolution at 6 tomograms per second, where only less than 0.3% of the beam intensity was utilized. Potentially, if the rotational stage is capable of faster rotations and the full beam intensity is utilized, the acquisition speed can reach 2000 tomograms per second, with the same effective pixel size of 4 µm, full dynamic range (12 bits) and 125 projections per tomogram.

## 5. Discussion and conclusion

We have studied the time-resolved 2D and 3D imaging capabilities down to the micrometre scale on the ForMAX beamline of MAX IV, the first operational DLSR.

Regarding time-resolved 2D imaging, we have demonstrated the potential to achieve frame rates of up to 1.1 MHz using the full dynamic range of the detector (12 bit) and an effective pixel size of 4 µm. This frame rate is comparable with state-of-the-art results (Olbinado *et al.*, 2017 ▸; Campbell *et al.*, 2021 ▸; Gao *et al.*, 2022 ▸). However, the reader must note that the results from ESRF (France) and APS (USA) can be obtained using a single pulse of those large storage rings. Small storage rings tend to have higher pulse frequencies, *e.g.* MAX IV has a 100 MHz pulse frequency. Thus, we are integrating approximately 90 pulses at the 1.1 MHz acquisition rate. This may blur dynamic effects, such as transient states, that happen on much faster temporal scales compared to single-shot approaches as implemented in large storage rings (Luo *et al.*, 2012 ▸; Olbinado & Rack, 2019 ▸) or X-ray free-electron lasers (Vagovič *et al.*, 2019 ▸). The key parameter to achieve a high temporal resolution with good contrast and resolution is the flux density. Based on ray-tracing simulations using *XRT* (Klementiev & Chernikov, 2014 ▸), the flux density is doubled at 12.7 keV compared with the case at 16.3 keV. As shown in Section 4.2.1, if the full beam intensity is used, a dynamic range of 12 bits can be achieved at 1.1 MHz (12.7 keV) or 550 kHz (16.3 keV). This complies with the fact that at the same dynamic range level, the limit of the frame rate is directly proportional to the flux density. Therefore, we could validate the capabilities of time-resolved 2D imaging on the ForMAX beamline by estimating acquisition rates that use the full dynamic range of our detector system, with the help of the high flux density provided by the 3 GeV DLSR at MAX IV.

Regarding time-resolved 3D imaging, we have demonstrated the potential to reach 2000 tps at full dynamic range when removing all filters. In such a scenario, we are mainly constrained by the rotation stage since we have to choose the acquisition rate based on the rotation speed. This means that we have to constrain ourselves to the full dynamic range by implementing filters to avoid saturating our detector. If we could reach higher rotation speeds, we could also increase our acquisition rate and would not have to rely on the use of attenuators at that point. In our experiments, we were limited to an operational rotation speed of 180 revolutions per minute (corresponding to 6 tps). Although ForMAX will be capable of faster rotations in the near future with its recently implemented rotation stage (720 revolutions per minute; Nygård *et al.*, 2024 ▸), the flux density can in principle lead to even higher acquisition rates, such as ktps scales. However, such fast time scales come with several limitations, *e.g.* the sensitivity of samples to centrifugal forces induced by rotation. For instance, the rotation of a sample at 500 Hz can induce up to 1000 *g* force on the sample, as reported in recent work (Garcia-Moreno *et al.*, 2021 ▸). This implies that we need to consider alternative methods, such as X-ray multi-projection imaging (XMPI) (Villanueva-Perez *et al.*, 2018 ▸; Duarte *et al.*, 2019 ▸; Voegeli *et al.*, 2020 ▸; Villanueva-Perez *et al.*, 2023 ▸; Asimakopoulou *et al.*, 2024 ▸), as a potential solution to overcome these challenges if the studied sample demands it. Considering these concepts, it becomes feasible to broaden the scope of samples suitable for examination on ForMAX and other future imaging beamlines at DLSRs. This encompasses not only samples that exhibit faster dynamics but also more complex sample systems where rotation is not possible. Fluid samples, such as blood, serve as an illustration of this scenario, opening up exciting prospects for conducting volumetric studies.

Apart from assessing the spatiotemporal capabilities in three dimensions, we have also demonstrated the successful acquisition of time-resolved 3D images of radiation damage induced on a wooden rod. Radiation damage plays a crucial role, as it is the ultimate limit in X-ray imaging experiments. On one hand, imaging experiments require a minimum dose to achieve a signal-to-noise ratio suitable for imaging at a given resolution. On the other hand, there is a limitation given by the maximum tolerable dose (Howells *et al.*, 2009 ▸). Being able to study the time scales on which radiation damage occurs can therefore help in improving the understanding of its dynamics, and ultimately in designing experiments that preserve the resolution not only for a given dose but a given dose rate. As one expects, the dose rate also plays a role in limiting the achievable resolution and not only the dose. Such studies are out of the scope of this paper.

In conclusion, we have successfully evaluated the time-resolved capabilities of the ForMAX beamline (MAX IV). The high flux densities of DLSRs make it possible to achieve higher temporal resolution without compromising on spatial resolution and using the full detector dynamic range, relaxing sample-contrast constraints. Furthermore, by using a single harmonic of the undulator spectrum, quantitative analysis becomes feasible compared to broader beam approaches when using a white beam.

MAX IV is not the only synchrotron light source enabling time-resolved imaging in the manner executed in this work. The crucial factor lies in the increased flux density provided by MAX IV and other DLSRs. Consequently, other storage rings that have undergone or are presently undergoing upgrades to DLSRs (*e.g.* ESRF-EBS, APS) offer or will offer comparable opportunities.

We have demonstrated that our experiments were not limited by the beam properties but rather by the instrumentation. Among the instrumentation limitations, the constraint given by rotation speed indicates the need to explore alternative approaches in order to study faster dynamics in three dimensions. A more efficient scintillator, and faster and more efficient detection schemes with smaller pixels, would help to improve the spatiotemporal resolution.

As a result of these benchmarking experiments and the increased availability of imaging beamlines at DLSRs, we envision exciting times for time-resolved imaging, which will open up new opportunities to address novel scientific questions by enabling new spatiotemporal resolutions.

## Figures and Tables

**Table 1:** Summary of the studied samples and experimental parameters in time-resolved 2D and 3D imaging

	Time-resolved 2D imaging	Time-resolved 3D imaging
Sample	Cellulose filaments (i)	Wooden rods
	Cellulose foams (ii)	
Sample dimension (l × w × h)	250–300 m × 250–300 m × 10 mm (i)	2 mm × 2 mm × 50 mm
	1 mm × 1 mm × 10 mm (ii)	
Dynamic process	Tensile loading (i)	Radiation damage
	Compression (ii)	
Energy	16.3 keV	9.1 keV
Effective pixel size	4 µm	4 µm
Acquisition frame rate	36 kHz	0.75 kHz
Imaging regime	Absorption	Absorption
